# Data for atmospheric arsenic deposition: A case study- northeast of Iran

**DOI:** 10.1016/j.dib.2018.05.119

**Published:** 2018-05-25

**Authors:** Zahra Atarodi, Javad Alinezhad, Reza Amiri, Yahya Safari, Nasrin Yoosefpour

**Affiliations:** a Student of Environmental Health Engineering, Health Faculty, Students Research Committee, Gonabad University of Medical Sciences, Gonabad, Iran; b Graduate Health Education and Health Promotion Center, Torbat Heydariyeh University of Medical Sciences, Torbat Heydariyeh, Iran; c Students Research Committee, School of Public Health, Kermanshah University of Medical Sciences, Kermanshah, Iran; d Department of Radiology, Paramedical School, Kermanshah University of Medical Sciences, Kermanshah, Iran

**Keywords:** Atmospheric arsenic, Air pollution, Air particle, PM_10_, Gonabad city

## Abstract

Air pollution is the major health concern in modern societies, especially in countries with arid and aggressive climate. Nowadays extensive research has been carried out to identify air pollution and its control. The main aim of this study is determine the atmospheric arsenic deposition concentration in Gonabad County in northeast Iran. In this cross-sectional study, the concentration of arsenic was measured by collecting of PM_10_ deposition from the ambient air of Gonabad urban areas. Samples were firstly taken by jar test method in four one-month periods in 2016 from Taleghani st., Imam Khomeini sq., Mend sq., Ghaffari st., and Sadi st., and arsenic concentration in the particles were determined by the Graphite furnace atomic absorption spectroscopy (GFAAS). The results indicated that the maximum and minimum concentrations (average) of particles PM_10_ depositing was observed in Taleghani st. about 10.395 ± 1.183 µg/kg and Imam Khomeini sq. about 4.394 ± 0.961 µg/kg, respectively. The maximum and minimum concentration of arsenic concentrations were estimated to be respectively 12.080 and 3.560 µg/kg in December and September, respectively. The results showed that in the northern part of the city, due to the wind blow, there are more particles in the air and people living in these areas are more exposed to arsenic. Therefore, residents of these areas need more actions that are preventive.

**Specifications Table**

**Table:** 

Subject area	Environmental science
More specific subject area	Chemistry
Type of data	Tables
How data was acquired	In this study, the Samples were taken from Gonabad city, with the distance of 3–5 km of each sampling station and 20 m away from buildings and sources of pollution. Arsenic concentration was measured by Graphite furnace atomic absorption spectroscopy (GFAAS) model 20AA, with graphite furnace Varian GTA-95 in the wavelength of 237.8 nm. Depositing particles weight was determined in accordance with the measurement method TDS in the textbooks.
Data format	Raw, analyzed
Experimental factors	The nitric acid was used for the acid digestion of samples. In addition, to improve the reading accuracy, 5.0 μg of nickel nitrate per liter was also added to the digested samples.
Experimental features	All methods of sampling and arsenic analysis of collected samples were performed according to the standard method presented in valid references.
Data source location	Gonabad city, Iran
Data accessibility	Data are included in this article

**Value of the data**

• Atmospheric fine particles are considered as one of the ambient air pollutants that can cause many problems for human health [1], [2], [3], [4], [5], [6], [7], [8], [9]. The data of this study investigates one of the most dangerous contaminants (Arsenic) that can be absorbed by human respiratory system through fine particles.
• In the study area (Gonabad city), a similar study has not been done so far, so data from this study can show atmospheric arsenic deposition in this area.
• In Iran, due to limited information in this field of research, the data of this study can be a useful basis for similar studies in other parts of Iran.
• The data showed that in the northern part of the Gonabad city, due to the wind blow, there are more particles in the air and people living in these areas are more exposed to arsenic. Therefore, residents of these areas need more actions that are preventive.

## 1 Data

The result of present study show that the maximum amount of particle depositing is 12.080 µg/kg in Taleghani st., and its minimum amount is 3.560 µg/kg in Imam Khomeini square. The maximum and minimum concentration of arsenic concentrations were estimated to be 12.080 and 3.560 µg/kg in December–January and September–November, respectively (Table 1).

**Table 1:** The mean concentration of arsenic in the particles.

**Sample number**	**Time period**	**Sampling stations**	**Final water, L**	**Arsenic, μg/L**	**TPD, mg**	**TPD, μg/Kg**
1	January	Ghaffari st.	0.815	3.3	89.48	5.602
2	December	Ghaffari st.	0.710	2.4	84.23	6.120
3	November	Ghaffari st.	0.625	2.6	31.43	7.450
4	September	Ghaffari st.	0.560	3.2	43.40	7.890
5	January	Imam Khomeini sq.	0.569	3.8	58.90	3.560
6	December	Imam Khomeini sq.	0.587	5.2	78.19	4.110
7	November	Imam Khomeini sq.	0.560	4.2	130.63	4.124
8	September	Imam Khomeini sq.	0.455	4.1	50.34	5.780
9	January	Mend sq.	0.443	7.3	88.40	4.984
10	December	Mend sq.	0.439	7.6	45.12	5.200
11	November	Mend sq.	0.429	8.1	154.34	5.711
12	September	Mend sq.	0.820	7.2	100.40	6.825
13	January	Sadi st.	0.745	3.1	36.35	7.673
14	December	Sadi st.	0.703	2.3	63.1	8.233
15	November	Sadi st.	0.655	3.1	29.24	8.689
16	September	Sadi st.	0.569	2.2	122.75	9.057
17	January	Taleghani st.	0.560	4.3	11.44	9.320
18	December	Taleghani st.	0.495	3.2	60.22	9.980
19	November	Taleghani st.	0.480	4.2	44.29	10.200
20	September	Taleghani st.	0.453	3.5	58.10	12.08

Minimum, maximum, and average amount of arsenic concentration existing in TPD for each sampling station in the whole period is shown in Table 2.

**Table 2:** The descriptive parameters re lasted to concentration of arsenic in different sampling stations (μg/kg).

**Sampling station**	**Minimum**	**Maximum**	**Mean ± SD**
Sadi st.	7.673	9.057	8.419 ± 0.597
Mend sq.	4.984	6.825	5.680 ± 0.822
Imam Khomeini sq.	3.560	5.780	4.394 ± 0.961
Ghaffari st.	5.602	7.890	6.766 ± 1.081
Taleghani st.	8.689	12.080	10.395 ± 1.183

Comparing arsenic concentration in different sampling periods and stations indicated that there is no significant difference between the arsenic concentration, and different sampling periods and stations (*P* > 0.05). The study results show that there is a significant correlation between the monthly average amount of TPD mg and its content׳s arsenic concentration μg/Kg (ASc), according to the Eq. (1).

(1) $$\text{TPD}=0.1106\times \text{ASc}-0.2304\;\;\;\;(R^{2}\;=\;\text{0}.94)$$

## 2 Study design, materials and methods

The Samples were taken from Gonabad city by jar method, with the distance of 3–5 km of each sampling station and 20 m away from buildings and sources of pollution, in the second half of the year 2016, during the four one-month periods in stations of Taleghani st., Imam Khomeini sq., Mend sq., Ghaffari st., and Sadi street. In the jar method, the flow contain particles passed from very fine pores filter by a vacuum pump jar at a time interval and at the end weight of it measured before and after processing (EPA sampling of ambient air, method IO-2.2). A digital scale measured particles mass with the accuracy of 0.0001 g. Arsenic concentration was measured by Graphite furnace atomic absorption spectroscopy (GFAAS) model 20AA, with graphite furnace Varian GTA-95 in the wavelength of 237.8 nm [10]. Depositing particles weight was determined in accordance with the measurement method TDS in the textbooks. Nitric acid was used for the samples acid digestion. To improve the reading accuracy, 5.0 micrograms of nickel nitrate per liter was also added to the digested sample [10], [11], [12], [13], [14], [15]. Mass amount of total particle depositing (TPD) was calculated by Eq. (2) in mass/mass percent:

(2) $$\mathrm{TDF}=\frac{∆m}{A}\times \frac{V_{1}}{V_{2}}$$

In which, ∆m is the crucible mass difference before and after exposure to incubator, $\frac{V_{1}}{V_{2}}$ is the proportion of the initial liquid volume to the liquid volume delivered to the crucible (volume factor), and A is the sampling container opening area.

All the necessary chemicals have been used here has been provided by Merck Germany. Water was distilled twice, and its electrical conductivity was less than 1.0 m mouse (mm), which was used in order to provide standard solutions, reagents, and washing dishes. Glass containers with an opening diameter of 15 cm and height of 30 cm were used for sample preparation.
