# Supplementary material for: Data for atmospheric arsenic deposition: A case study- northeast of Iran
Source: Data Brief. 2018 May 25;19:660–4. doi: 10.1016/j.dib.2018.05.119 (PMC5997950; doi:10.1016/j.dib.2018.05.119)
Supplement: Supplementary file 1 — Transparency document [file mmc1.docx]

Dear editor(s)

Data in Brief

At this time, this article with entitled” ***Data for atmospheric arsenic deposition: A case study- northeast Iran*** “has not been published or submitted in any other journals. We hope that the results of this research will be of interest to you. None of the authors of this paper have any conflicts of interest to declare.

Best regards

Corresponding author

Nasrin Yoosefpour
